# Massive lung collapse with partial resolution after several years: a case report

**DOI:** 10.1186/1471-2431-5-39

**Published:** 2005-10-21

**Authors:** Elke Govaere, Dirk Van Raemdonck, Hugo Devlieger, Maria-Helena Smet, Eric Verbeken, Marijke Proesmans, Kris De Boeck

**Affiliations:** 1 University Hospital of Leuven, Dept of Pediatrics, Herestraat 49, 3000 Leuven, Belgium; 2 Dept Thoracic Surgery, Herestraat 49, 3000 Leuven, Belgium; 3 Dept Radiology, Herestraat 49, 3000 Leuven, Belgium; 4 Dept of Pathology, Herestraat 49, 3000 Leuven, Belgium

## Abstract

**Background:**

Bronchitis obliterans is a severe and extremely rare complication of respiratory tract infections in children and is characterized by massive atelectasis and collapse of the affected lung. Of the rare cases reported in the literature all surviving children underwent surgical resection of the collapsed lung.

**Case presentation:**

We report an infant with bronchitis obliterans that was treated conservatively. 5 years after the initial event, partial lung re-expansion was documented.

**Conclusion:**

This case therefore supports a conservative treatment whenever possible with pneumonectomy only as a last treatment option.

## Background

In children bronchiolitis obliterans occurs most commonly secondary to infection [1]. Rarely the fibroblastic obliteration involves the more central, cartilage-containing bronchi, leading to *bronchitis *obliterans, characterised by massive atelectasis and collapse of the affected lung. There are only five case reports of *bronchitis *obliterans [2-6]. All patients underwent surgery except one who died early in the course. We report a case of obliterative *bronchitis *without bronchiolar involvement following bilateral bronchopneumonia in a 9-month-old boy. Massive lung collapse occurred with partial spontaneous re-expansion 5 years later. To our knowledge, this is the first case report documenting such an evolution. Therefore, if possible, initial conservative treatment should be considered.

## Case presentation

AS was born by caesarean section at the postmenstrual age of 38 weeks. At the age of nine months, he was admitted to a regional hospital with cough, high fever and moderate tachypnea. Chest X-ray revealed bilateral pulmonary infiltrates and the child was treated with intravenous antibiotics. Ten days later he was referred to the university hospital because of persistent cough, increasing respiratory distress and atelectasis of the right lower and middle lobes on chest X-ray.

At referral we saw a pale and dystrophic child in moderate respiratory distress. Weight was on the third percentile for a height on the 75th percentile. The child was afebrile. He had chest hyperinflation, tachypnea (60 per minute) and intercostal retractions. Fine rales were heard over the right hemithorax. Transcutaneous oxygen saturation was 92% when awake but dropped below 90% during sleep. White blood cell count was 22900 per microliter (42% neutrophils), C-reactive protein was slightly elevated at 25 mg/L (normal value below 5 mg/L). Bronchoscopy demonstrated patent central airways: no mucus plug, no foreign body, no external airway compression. There was some erythema of the mucosa and scanty secretions in the right bronchial tree. Neutrophils (90%; normal less than 20%) predominated in the bronchoalveolar lavage fluid. *Haemophilus influenzae *and *Moraxella cattharalis *were isolated from the bronchial aspirate; no fungi nor mycobacteria were isolated. Viral cultures and/or paired serology for respiratory syncytial virus, cytomegalovirus, adenovirus, influenza, parainfluenza and *Mycoplasma pneumoniae *remained non-diagnostic. Serum immunoglobulins were within the normal range for age. The sweat chloride was 13 mmol/l. (normal <40 mmol/L).

Treatment with intravenous cefotaxime, supplemental oxygen and intensive chest physiotherapy was initiated but total right lung atelectasis, shift of the mediastinum to the affected side and herniation of the left lung into the right hemithorax was obvious on chest CT scan. Several small areas of ground glass opacities and overexpansion were present in the left lung. Only mild respiratory distress persisted, despite obvious shift of trachea and left ictus to the right and bronchial breath sounds over the entire right hemithorax. Transcutaneous oxygen saturation in room air was normal. On ventilation-perfusion scan there was no perfusion nor ventilation to the right lung but the left lung appeared normal. Cardiac angiography showed displacement of the heart towards the right hemithorax, normal anatomy of heart and large vessels and very slow circulation in the right pulmonary artery.

Several non-invasive as well as invasive interventions were done to recruit ventilation of the right lung: oral steroids (dexamethasone 0.6 mg/kg during 1 week), bronchial instillations of 5% acetyl-cysteine (3 × 20 ml), selective intubation and ventilation into the right main bronchus during 1 hour, instillation of surfactant (Survanta 200 mg). Total right lung collaps persisted. Cefotaxime was administered intravenously for a total duration of 3 weeks; thereafter amoxicillin was continued orally for 2 weeks despite negative lower airway cultures during repeat bronchoscopy and during selective intubation. A puncture biopsy of the right lung was performed. Histology revealed a thickened visceral pleura due to fibrosis and collapsed normal lung without signs of fibrosis nor inflammation. Ultrastructural examination of the cilia excluded primary ciliary dyskinesia. Thoracoscopy was performed to exclude lung entrapment by the pleural thickening and revealed a normal pleural cavity and a collapsed right lung. A right lower lobe biopsy showed collapsed lung with normal histology and a slightly thickened visceral pleura and interlobular septae. Absence of abnormalities in the terminal and respiratory bronchioles excluded bronchiolitis obliterans. Post thoracotomy, again in an effort to gain lung expansion, the boy was ventilated using high frequency oscillation for three days during which a second fruitless trial with selective intubation was performed.

After 6 weeks the patient was discharged with a residual mild tachypnea (35/minute) and retractions as well as bronchial breathing over the right hemithorax. Transcutaneous oxygen saturation was 98%. The further evolution was rather favourable. During winter mild lower respiratory infections and episodes of nocturnal cough treated with oral antibiotics occurred. Exercise tolerance was reported as 'comparable with children his age'. From the age of 5 years on an obvious chest asymmetry with smaller right hemithorax is observed in addition to a mild scoliosis. Spirometry at the age of 6 years shows a mixed restrictive and fixed obstructive pattern: total lung capacity 75% predicted, forced vital capacity 69% predicted, forced expired volume in one second 57% predicted.

**Figure 1:**
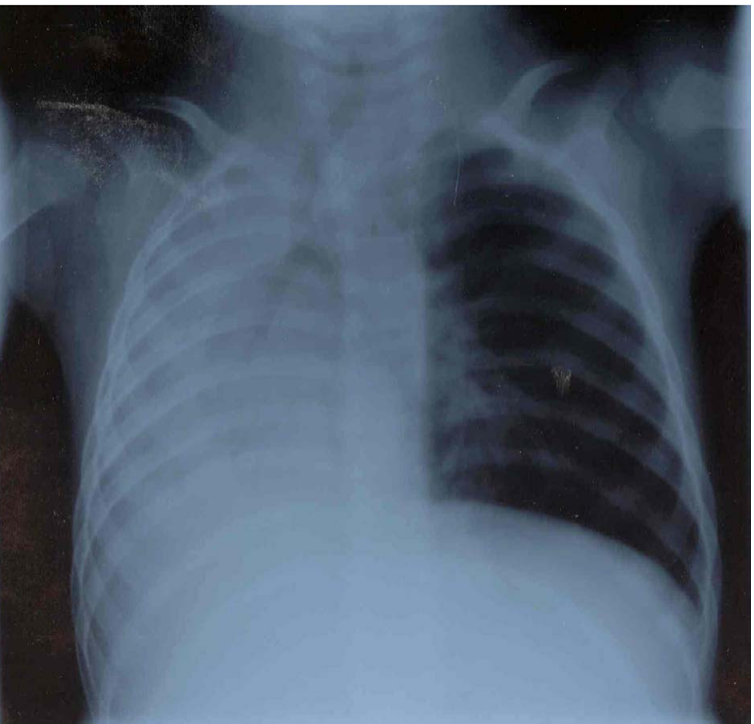
Chest X-ray shows persisting total atelectasis of the right lung. Note the marked loss of volume on the right, pronounced shift of the mediastinum to the right, and compensatory overexpansion of the left lung.

**Figure 2:**
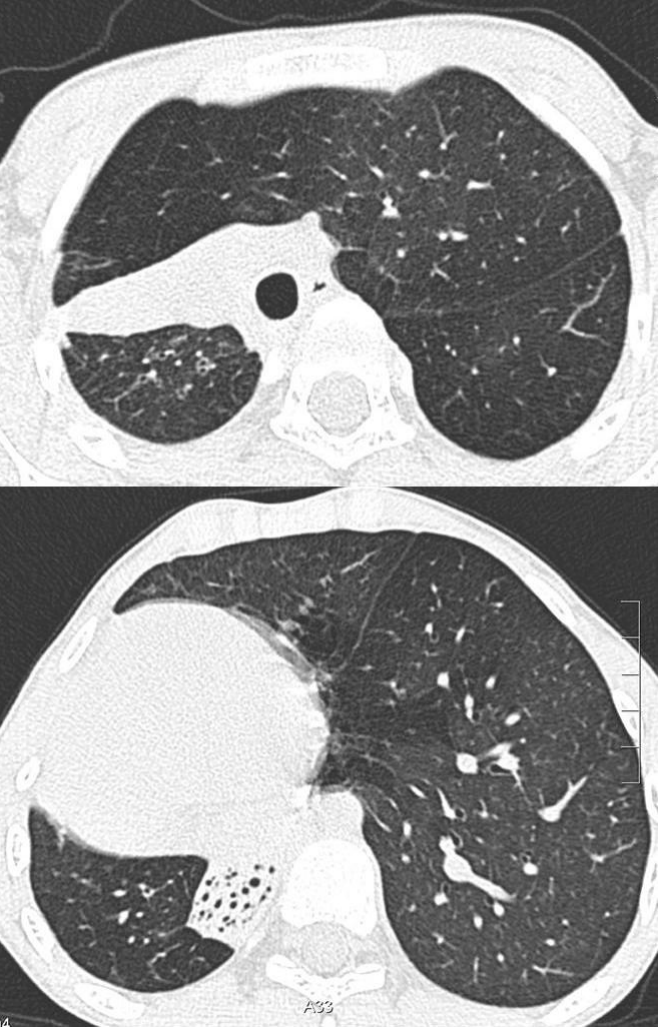
A High resolution computed tomography demonstrates partial re-expansion of the right lung with very moderate bronchiectasis. B The right lower lobe remains collapsed and shows more extensive bronchiectasis.

Total right lung collapse documented radiologically persisted (fig 1). But at the age of 6.5 years, a high resolution computed tomography scan showed a re-expansion of the right upper and middle lobes with bronchiectasis in these lobes (fig 2). The right lower lobe remains collapsed with part of the left lung herniated into the right hemithorax. This partial re-expansion 5 years after the insult is associated with minute improvement in spirometric values: FVC 74% predicted and FEV_1 _62% predicted). Although no formal exercise testing was performed exercise capacity is very good since the patient was second in his school athletic competition run.

## Discussion

Histologically, bronchiolitis obliterans is defined by obliteration of the lumen of bronchioles due to inflammation, granulation tissue or scarring [1]. The most common occurrence of bronchiolitis obliterans in children is postinfectious: adenovirus, respiratory syncytial virus, influenza, measles and *Mycoplasma pneumoniae *[1,8]. High-resolution computed tomography shows mostly hyperinflation, bronchial wall thickening and mosaic patterns, areas of bronchiectasis and atelectasis. Unilateral collapse is rather rare [9]. The definitive diagnosis of bronchiolitis obliterans was traditionally based upon histopathological confirmation. It should however be strongly suspected based on clinical and radiologic findings, i.e. more than 6 weeks of continuous wheezing, cough and tachypnea following an acute bronchiolitis associated with radiological changes. The necessity of lung biopsy to make the diagnosis is under debate [8,10].

Relative to obliterative bronchiolitis, obliterative *bronchitis *due to obliteration of the segmental and subsegmental bronchi without bronchiolar involvement is rare. Unilateral pulmonary collapse is the most frequent presentation. The pathogenesis is uncertain [11]. Only five reports describe *bronchitis *obliterans [2-6]. All children underwent pneumonectomy except one patient who died early in the course. Two cases were presumed to be of viral etiology [4,6], one case was related to *Mycoplasma pneumoniae *[2] infection. Two other cases were associated with Stevens-Johnson syndrome [3] and hypogammaglobulinemia [5]. We describe a first case of *bronchitis *obliterans treated conservatively in whom partial lung re-expansion occurred five years after the onset of disease.

Our case, a previous healthy 9-month-old boy, developed a severe respiratory distress with collapse of the right lung, following a bilateral bronchopneumonia. Microscopic examination demonstrated normal collapsed lung tissue that was histologically normal and slightly thickened visceral pleura and interlobular septa. No abnormalities of the membranous and respiratory bronchioles were identified, so the diagnosis of bronchiolitis obliterans can be excluded. The central cartilaginous bronchi were not present in the biopsy, so the diagnosis of *bronchitis *obliterans was made on clinical and radiological ground but could not be confirmed histologically since no lung resection was performed. However, what other diagnosis would be possible when bronchiolitis obliterans is excluded, a foreign body, local airway compression and mucus plug are ruled out and the lung collapse is total and resistant to selective intubation and ventilation (even supplemented with surfactant instillation and steroid treatment).

Because of relatively mild respiratory symptoms and the risk of a post-pneumonectomy syndrome [12], a conservative approach was maintained. To our surprise, five years after the initial diagnosis, a partial re-expansion of the affected right lung was noted on high-resolution computed tomography. This is the first case ever described with a comparable evolution. Could recanalization of larger airways be the cause? Reversibility of pulmonary involvement is documented in *bronchiolitis obliterans organising pneumonia *(BOOP) and other diseases like idiopathic interstitial pneumonia but has not been described in bronchitis obliterans.

## Conclusion

This case illustrates that for children with bronchitis obliterans, surigical resection of the collapsed lung may not always be necessary. In our opinion, conservative treatment should be considered when possible. Pneumonectomy or lobectomy should only be reserved as a last option.

## List of abbreviations

BOOP: *bronchiolitis obliterans *organising pneumonia

## Competing interests

The author(s) declare that they have no competing interests.

## Authors' contributions

Clinicians responsible for patient management: M. Proesmans, E. Govaere, K. De Boeck

Surgeon who performed surgery: D. Van Raemdonck

Intensivist: H. Devlieger

Radiologist: M.H. Smet

Pathologist: E. Verbeken

## Pre-publication history

The pre-publication history for this paper can be accessed here:


